# Survey of ICU nurses’ cognition, attitude, and practice on acute skin failure

**DOI:** 10.1097/MD.0000000000042237

**Published:** 2025-09-05

**Authors:** Xiuhong Lu, Yanlan Lu, Shuangwen Qin, Ling Li, Debin Huang

**Affiliations:** a Department of Critical Care Medicine, The First Affiliated Hospital of Guangxi Medical University, Nanning, China; b Department of Critical Care Medicine, Guangxi Hospital Division of the First Affiliated Hospital, Sun Yat-sen University, Guangzhou, China.

**Keywords:** ASF, attitude, cognition, intensive care unit, practice

## Abstract

This study aims to investigate the cognition, attitude, and practice of acute skin failure (ASF) among nurses in the intensive care unit (ICU) and analyze its influencing factors. In July 2023, an ASF Cognition Status Questionnaire was used to survey 370 ICU nurses via a convenience sampling method from 12 hospitals in Guangxi Province, China. The total score for the ASF Cognitive Status Questionnaire was (100.95 ± 20.63), with scores of (23.15 ± 10.96), (37.72 ± 4.75), and (40.08 ± 10.82) for the cognition dimension, attitude dimension, and practice dimension, respectively. Multiple linear regression analysis revealed that years of work experience, membership on a skincare team, have received hospice care training, and hospital grade were significant factors affecting ICU nurse’s level of ASF knowledge and practice proficiency. ICU nurses’ overall cognitive status level of ASF is low, and their attitude dimension and practice dimension are much better than their cognition dimension. Nurses with shorter working years, belong to skincare team, having completed hospice training, and working at higher grade hospital exhibit greater nursing cognitive status about ASF. Nursing managers should focus on the training of ICU nurses’ ASF-relevant knowledge, and take corresponding training measures for different groups of nurses to improve ICU nurses’ cognitive status of ASF.

## 1. Introduction

As the largest organ of the human body, skin serves as the primary barrier against various pathogenic microorganisms.^[1]^ Acute skin failure (ASF) is generally characterized by hypoperfusion-induced damage to skin tissue, commonly observed in critically ill patients and often occurring towards end-of-life stages.^[2–5]^ Skin management plays a crucial role in the intensive care unit (ICU). Due to their similar appearance, ASF and pressure injury (PI) are frequently misidentified.^[6]^ PI is a nursing adverse event that has garnered significant attention clinically. The potential for misdiagnosis or missed diagnosis of ASF not only hampers clinical nursing quality management but also leads to medical disputes^[3]^ and increases additional economic burdens on patients and healthcare institutions. As frontline caregivers responsible for skincare, ICU nurses have extensive patient interaction time, thus, their understanding and proficiency in identifying, evaluating, and managing ASF significantly impacting the quality of skincare provided. Currently, there are few reports about the ICU nurses’ cognition status of ASF. This study aims to investigate ICU nurses’ cognition status of ASF, provide data support and improvement suggestions for future training programs, and enhance nurses’ ability to identify and respond to ASF.

## 2. Methods

### 2.1. Survey respondent

In July 2023, 370 ICU nurses were selected using convenience sampling method from 12 hospitals in Guangxi, China. Inclusion criteria included practicing qualification certificate-holding on-the-job nurses with a minimum of 1-year experience in the ICU. Exclusion criteria encompassed advanced students, standardized rotation nurses, and nursing students.

### 2.2. Survey tools

#### 2.2.1. General information questionnaire

This encompasses variables such as gender, Professional level, years of professional experience, educational background, membership in the skincare team, type of ICU, receive hospice training, and hospital grade.

#### 2.2.2. ICU nurses’ questionnaire of ASF cognitive status

Compiled by Yang et al.,^[7]^ the questionnaire includes 33 items in 3 dimensions. (1) Cognition section, a total of 11 items. All the items are scored by Likert 5-level scale, which are divided into 5 grades: “Don’t know,” “heard of,” “generally understand,” “relatively understand,” and “very understand,” and give 1 to 5 points, respectively. The total score of this dimension ranges from 11 to 55 points, and the higher the score, the higher the degree of cognition of ASF. (2) Attitude section, a total of 10 items. All the items are scored by Likert 5-level scale, which are divided into 5 grades: “strongly disagree,” “disagree,” “uncertain,” “agree,” and “strongly agree,” and give 1 to 5 points, respectively. The total score of this dimension ranges from 10 to 50 points, and the higher the score, the better the attitude of ICU nurses toward ASF nursing. (3) Practice section, a total of 12 items. All the items are scored by Likert 5-level scale, which are divided into 5 grades: “never,” “occasionally,” “sometimes,” “often,” and “always,” and give 1 to 5 points, respectively. The total score of this dimension ranges from 12 to 60 points, and the higher the score, the higher the level of practice of ICU nurses toward ASF. Cronbach’s α coefficient of the total questionnaire was 0.941, and the content validity index was 0.848. The Cronbach’s α coefficients of cognition, attitude, and practice were 0.923, 0.932, and 0.934, respectively. The results indicate that the questionnaire has good reliability and validity.

### 2.3. Data collection methods

In this study, the Wenjuan Star online platform questionnaire was utilized for data collection. The 2-dimensional code of the questionnaire was distributed to relevant departments within the investigation hospital, accompanied by detailed instructions on how to complete each section. Furthermore, it was emphasized that nurses should refrain from consulting pertinent literature and independently fill out the questionnaire. All questions listed in the questionnaire were mandatory, and each electronic device could only submit 1 response. A total of 398 questionnaires were distributed in July 2023. However, any questionnaires with identical responses and completion times less than 3 minutes were excluded, resulting in a final collection of 370 questionnaires.

### 2.4. Data analysis

Data were analyzed using SPSS Statistics software (Version 23.0). In univariate analysis, *t* tests or analysis of variance test was used to analyze the differences in cognition, attitude, and practice scores of ICU nurses with different characteristics on ASF, and multiple linear regression was used to analyze the related influencing factors.

## 3. Results

### 3.1. General information of survey respondents

Among 370 ICU nurses, 46 (12.43%) were male and 324 (87.57%) were female. Professional titles include 208 junior nurses (56.22%), 115 Supervisor nurses (31.08%), and 47 deputy chief nurses and above (12.7%). Working years include the following: 104 nurses (28.11%) in 1 to 5 years, 126 nurses (34.05%) in 6 to 10 years, 106 nurses (28.65%) 11 to 20 years, and 24 (9.19%) more than 20 years. Education details are follows: junior college degree 111 (30%), undergraduate 256 (69.19%), and Master 3 (0.81%). There were 48 members (12.97%) in the skincare group, and 322 members (87.03%) in non-skincare group. There were 324 nurses (87.67%) in comprehensive ICU and 46 nurses (12.33%) were in other ICUs. Twenty-eight ICU nurses (7.57%) received hospice care training while 342 (92.43%) did not. A total of 233 nurses (62.97%) in tertiary hospitals and 137 nurses (37.03%) in secondary hospitals.

### 3.2. Questionnaire score and single-factor analysis of ICU nurses’ cognition of ASF

The total score of ICU nurses’ ASF cognitive status questionnaire was 100.95 ± 20.63 points, among which the score of cognition dimension was 23.15 ± 10.96, the score of attitude dimension was 37.72 ± 4.75 and the score of practice dimension was 40.08 ± 10.82. Univariate analysis showed that there were statistically significant differences in ASF cognition scores among ICU nurses of gender, professional title, working years, whether they were members of the skincare team, whether they received hospice care training, and hospital grade (*P* < .05). The scores of ASF attitude in ICU nurses of different hospital grades were statistically significant (*P* < .05). There were statistically significant differences in ASF practice dimension scores among ICU nurses whether have received hospice training, and working in different hospital grades (*P* < .05), as shown in Table 1. In addition, the 3 lowest scores items of the 3 dimensions of ASF are shown in Table 2.

**Table 1:** Univariate analysis of factors affecting ICU nurses’ ASF cognition, attitude, and practice.

	Number of people (percentage)	Cognition	Attitude	Practice
Gender				
Male	46 (12.43%)	26.85 ± 13.47	38.36 ± 5.59	40.71 ± 11.65
Female	324 (87.57%)	22.62 ± 10.48	37.62 ± 4.62	39.99 ± 10.71
*t*		2.043	0.864	0.042
*P*		.046	.391	.672
The title of a professional post				
Nurse	208 (56.22%)	24.71 ± 11.84	37.55 ± 4.50	40.14 ± 11.59
Supervisor nurse	115 (31.08%)	20.24 ± 9.21	37.82 ± 5.21	39.95 ± 9.58
Associate chief nurse and above	47 (12.7%)	23.32 ± 9.54	38.19 ± 4.72	40.17 ± 10.34
*F*		6.335	0.383	0.130
*P*		.002	.682	.987
Years of working experience				
1–5	104 (28.11%)	26.52 ± 11.59	37.59 ± 4.67	40.92 ± 11.99
6–10	126 (34.05%)	22.21 ± 11.20	37.61 ± 4.37	39.40 ± 10.62
11–20	106 (28.65%)	20.96 ± 9.79	37.92 ± 5.48	40.17 ± 10.22
>20	24 (9.19%)	23.09 ± 9.46	37.85 ± 4.00	39.06 ± 9.84
*F*		5.156	0.123	0.396
*P*		.002	.947	.756
Educational level				
Subdegree	111 (30%)	24.85 ± 12.34	37.75 ± 4.34	40.13 ± 11.45
Bachelor	256 (69.19%)	22.41 ± 10.25	37.66 ± 4.88	39.99 ± 11.45
Master	3 (0.81%)	23.33 ± 13.05	41.00 ± 8.72	46.33 ± 12.34
*F*		1.929	0.734	0.509
*P*		.147	.481	.601
Is a member of the skincare team				
Yes	48 (12.97%)	26.56 ± 11.06	38.46 ± 4.98	42.13 ± 10.45
No	322 (87.03%)	22.64 ± 10.87	37.61 ± 4.71	39.78 ± 10.86
*t*		2.328	1.161	1.403
*P*		.020	.247	.161
Type of ICU				
Comprehensive ICU	324 (87.67%)	23.20 ± 10.86	37.70 ± 4.64	40.15 ± 10.59
Other ICU	46 (12.33%)	22.76 ± 11.78	37.83 ± 5.54	39.63 ± 12.41
*t*		0.245	0.167	0.303
*P*		.799	.867	.762
Have received hospice training				
Yes	28 (7.57%)	35.64 ± 11.31	38.43 ± 3.84	44.50 ± 9.56
No	342 (92.43%)	22.12 ± 10.30	37.66 ± 4.82	39.72 ± 10.85
*t*		6.629	0.825	2.259
*P*		<.001	.410	.024
Hospital grade				
Tertiary hospital	233 (62.97%)	25.00 ± 11.73	38.18 ± 5.08	41.04 ± 10.96
Secondary hospital	137 (37.03%)	20.00 ± 8.70	36.93 ± 4.03	38.46 ± 10.42
*t*		4.674	2.593	2.226
*P*		<.001	.010	.027

ASF = acute skin failure, ICU = intensive care unit.

**Table 2:** Items with the lowest score of 3 in each dimension of ASF cognition, attitude, and practice of ICU nurses.

	Item	Score
Cognition	7. Do you know the evaluation method of skin perfusion pressure	1.91 ± 1.21
	2. Do you know the pathogenesis of acute skin failure	1.98 ± 1.04
	8. Do you know the evaluation tools for tissue perfusion	2.00 ± 1.13
Attitude	20. Do you think it is important to develop standardized procedures for the management of acute skin failure	2.47 ± 1.17
	21. Do you think acute skin failure is an inevitable condition for critically ill patients	2.57 ± 1.03
	14. Do you think acute skin failure is different from stress injury	3.82 ± 0.79
Practice	25 Whether you will evaluate the patient for skin perfusion	2.73 ± 1.27
	31. Do you record patients’ acute skin failure in a timely manner	2.81 ± 1.23
	29. Do you focus on assessing patients who have developed acute skin failure	2.82 ± 1.21

ASF = acute skin failure, ICU = intensive care unit.

### 3.3. Multivariate linear regression analysis of cognitive status questionnaire scores of ICU nurses on ASF

The 3 dimensions of ASF scores among ICU nurses were considered as dependent variables, while the variables demonstrating statistical significance in the results of univariate analysis were regarded as independent variables (including gender, professional titles, years of working experience, membership in the skincare team, receive hospice training, and hospital grade for conducting multiple linear regression analysis. The assignment of arguments was performed as follows: (1) Gender: male = 1, female = 2. (2) professional titles: nurse = 1, supervisor nurse = 2, deputy chief nurse and above = 3. (3) Years of working experience: 1 to 5 years = 1, 6 to 10 years = 2, 11 to 20 years = 3, >20 years = 4. (4) Membership in the skincare team: yes = 1, no = 2. (5) Receive of hospice training: yes = 1, no = 2. (6) Hospital grade: level 3 hospital = 1, level 2 hospital = 2. The findings revealed that factors influencing ICU nurses’ ASF scores include their years of working experience, membership in the skincare team, receiving hospice training, and hospital grade. Different ICU nurse populations, such as working years, whether they are members of the skin care team, whether they receive hospice care training, and hospital grade, have different perceptions of ASF, which are mainly reflected in the knowledge dimension. However, hospital grade alone exhibited statistically significant differences within both attitude and practice dimensions (as shown in Table 3).

**Table 3:** Multivariate linear regression analysis of factors affecting ICU nurses’ ASF cognition, attitude, and practice.

Dependent variable	Independent variable	*B*	Standard error	*ß*	*t*	*P*
Cognition	Constant	68.446	5.578	-	12.271	<.001
	Years of working experience	−1.721	0.866	−0.149	−1.988	.048
	Is a member of the skincare team	−4.087	1.611	−0.125	−2.537	.012
	Have received hospice training	−12.066	2.003	−0.291	−6.023	<.001
	Hospital grade	−4.469	1.084	−0.197	−4.122	<.001
Attitude	Constant	42.663	2.643	-	16.141	<.001
	Hospital grade	−1.327	0.514	−0.135	−2.584	.010
Practice	Constant	56.775	6.000	-	9.463	<.001
	Hospital grade	−2.445	1.166	−0.109	−2.097	.037

ASF = acute skin failure, ICU = intensive care unit.

## 4. Discussion

### 4.1. The overall score of ICU nurses’ cognition of ASF was not satisfactory, especially the level of cognition dimension was poor

The results revealed that the total score of ICU nurses for ASF was 100.95 ± 20.63, and the score rate was 60.61%. The score of cognition part was 23.15 ± 10.96, and the score rate was only 42.09%, which closely aligns with previous similar studies.^[8]^ These findings indicate that ICU nurses possess insufficient overall cognition regarding ASF, and their mastery of cognition is low. ASF was first proposed by La Puma^[9]^ in 1991. Although it has received the attention from clinical doctors and nurses, the research on ASF is still in the preliminary stage and lacks mature tools for targeted evaluation. In the Cognition entry, “Do you know the evaluation method of skin perfusion pressure (1.91 ± 1.21 points),” “Do you know the pathogenesis of ASF (1.98 ± 1.04 points),” “Do you know the evaluation tools of tissue perfusion (2.00 ± 1.13 points),” and “Do you know the definition of ASF (2.07 ± 1.03 points).” The score of cognition dimension was the lowest, which also was the lowest among all 33 items, indicating that ICU nurses lacked understanding of the definition and pathogenesis of ASF, and not familiar with the assessment tools and methods related to ASF. In addition, this study also found that although most nurses had a low cognition dimension, their scores on attitude and practice were much higher than those on cognition dimension, which was inconsistent with the results of previous studies on cognition, belief, and practice,^[8]^ resulting in the separation of cognition from attitude and practice. However, various corresponding trainings in the later stage could improve the cognition level of ICU nurses on ASF. Their attitude and practice will be improved accordingly, so it does not violate the theoretical model of cognition, attitude, and practice. To sum up, the manager should train ICU nurses with different characteristics through multiple channels and in various forms, explain the pathophysiological cognition of ASF in a simple way, and combine its specific clinical manifestations to make its understanding more profound. The relevant assessment tools and correct assessment methods^[10,11]^ are used to detect and identify ASF as early as possible, and timely nursing measures are taken to prevent it from happening.

### 4.2. ICU nurses with shorter years of working experience, members of the skincare team, and who have received hospice training demonstrate higher levels of cognition in ASF

#### 4.2.1. Years of working experience

This study shows that nurses with shorter years of working experience have a higher score on ASF cognition, which is inconsistent with Wang et al.^[12]^ We believe that the reasons may be as follows: First, younger nurses are more receptive to new things, more inclusive to new concepts, and more willing to take the initiative to learn new professional cognition. Second, with the development of nursing discipline in our country, nurses’ education is constantly improving. Younger nurses are mostly bachelor’s degree holders, and they are better than senior nurses at acquiring abundant ASF cognition through the Internet and other channels. The research also showed^[13]^ that nurses with lower working experience less occupational fatigue, less conflict between work and family life, more time to explore clinical doubts related to skin failure, and a more positive learning attitude. Therefore, managers should take targeted training measures according to the characteristics of nurses with different seniority. Nurses with long working years are encouraged to overcome their fear and resistance to the Internet, actively and boldly participate in online information retrieval, and obtain rich information resources, experienced nurses in the department were invited to answer questions and provide relevant support.

#### 4.2.2. Skincare team members

This study showed that ICU nurses who were members of the skincare team had better cognition of ASF, which was consistent with the results of Yang et al.^[14]^ Skincare team nurses participate in skin specialist training more frequently and have more opportunities and ways to learn ASF-related cognition. Although ASF and PI are similar in appearance, nurses trained in skincare can identify the difference, early and correct identification of ASF is a prerequisite for taking effective measures. In this study, only 48 (12.97%) of the 370 ICU nurses were members of the skincare team, indicating that there were too few nurses with professional training in skin to meet clinical care needs. It is recommended that managers increase the number of skin specialist nurses, so that each ICU team has at least one member of the skincare specialist nurse. Give full play to its leading role to ensure that there are specialized nurses for dynamic observation and nursing to ensure the quality of care for ASF patients.

#### 4.2.3. Trained in hospice care

Previous studies have shown that^[11]^ the incidence of ASF is as high as 83.7% in ICU, and the occurrence of ASF is closely related to the prognosis of patients. Patients with ASF have high mortality and poor prognosis. Most ICU patients are in critical condition and are extremely prone to ASF at the end of life. ASF brings tremendous impact on the senses and psychological damage to the families of terminal patients. This study shows that ICU nurses who have received hospice training have a higher cognition level of ASF, indicating that nurses who have received hospice training have more caring ability and humanistic care spirit,^[15]^ pay more attention to skin failure of patients, attach importance to skin management of patients, provide psychological support to families of patients, and improve the quality of death of patients.^[16]^ It also avoids unnecessary medical expenses.^[17]^ This study also showed that only 28 (7.57%) ICU nurses had received hospice care training, indicating the popularization of hospice care training in ICU nurses is not optimistic, and suggesting managers should fully popularize hospice care training to improve the quality of end-of-life care for ICU nurses.

#### 4.2.4. Hospital grade

This study shows that hospital grade is the main influencing factor of ICU nurses’ cognition, attitude, and practice toward ASF, which is consistent with the results of Wang et al^[12]^ and Wei et al.^[18]^ The reasons may be as follows: On the one hand, the higher the hospital grade, the more attention the nursing managers pay to training. Nurses are constantly strengthened in training, their theoretical cognition level is improved, and their attitude and practice level are also improved. On the other hand, tertiary hospitals have a strong academic research atmosphere, rich academic platform resources, and a good learning environment that makes nurses more interested in ASF-related cognition, more motivated to learn, and more critical thinking about clinical problems. Therefore, we should pay attention to the training of nurses in secondary hospitals and actively provide them with a learning platform. It is suggested that secondary hospitals send more nurses to higher hospitals for further study to expand their cognition. At the same time, establish and improve the remote skin consultation network platform to provide consultation guidance, form a virtuous circle of positive feedback, and improve the level of cognition, attitude and practice of ICU nursing staff ASF in secondary hospitals from all aspects.

## 5. Conclusions

In this study, 12 hospitals in Guangxi Zhuang Autonomous Region were selected to carry out a cross-sectional survey. The results showed that years of working time, whether they were members of the skincare team, whether they received hospice care training, and hospital grade are the influencing factors for ICU nurses’ cognition of ASF, which could provide a certain reference for nursing managers to take targeted training. Nursing educators and managers should pay attention to carrying out ASF nursing training for ICU nurses, improve nurses’ cognition and attitude of ASF, promote the implementation of ASF-related nursing practice, and improve ICU patient safety and nursing quality.

### 5.1. Limitations

However, due to geographical restrictions and sample method, this study has some limitations. Convenient sampling makes the results possibly less representative. In the future, it is possible to carry out multicenter investigations in several provinces and cities across the country, so as to have a more comprehensive understanding of ICU nurses’ cognition of ASF.

## Author contributions

**Data curation:** Xiuhong Lu, Shuangwen Qin, Ling Li.

**Project administration:** Xiuhong Lu, Debin Huang.

**Validation:** Xiuhong Lu.

**Writing–original draft:** Xiuhong Lu.

**Supervision:** Yanlan Lu, Debin Huang.

**Writing–review & editing:** Yanlan Lu, Debin Huang.

**Investigation:** Shuangwen Qin, Ling Li.
